# Molecular Diagnosis of Syphilis in Brazilian Ambulatory Patients: Detection of *Treponema pallidum* subsp. *pallidum* in Serum Using Ancient DNA Protocols

**DOI:** 10.3390/microorganisms14020453

**Published:** 2026-02-12

**Authors:** Lorrayne Samille Santos de Brito, Mauro Romero Leal Passos, Alena Mayo Iñiguez

**Affiliations:** 1 Laboratório de Parasitologia Integrativa e Paleoparasitologia, Instituto Oswaldo Cruz, Fundação Oswaldo Cruz (Fiocruz), Rio de Janeiro 21040-900, Brazil; lorraynebrito1992@gmail.com; 2 Setor de Doenças Sexualmente Transmissíveis, Departamento de Microbiologia e Parasitologia, Instituto Biomédico, Universidade Federal Fluminense, Rio de Janeiro 24210-150, Brazil; maurodst@gmail.com

**Keywords:** *Treponema pallidum*, molecular detection, serological samples

## Abstract

The rising incidence of syphilis in recent decades underscores the need to improve diagnostic and control strategies. The infection caused by *Treponema pallidum* subsp. *pallidum* is commonly diagnosed using serological tests. However, these methods exhibit limitations in the early or late stages of disease, when antibody responses and/or bacterial loads are low. Molecular biology detection using serum samples is also hampered by low circulating bacterial loads during asymptomatic periods. Ancient DNA (aDNA) studies apply methods adapted to recovering low concentrations and degraded DNA. In this study, we evaluated the effectiveness of aDNA protocols applied to the molecular diagnosis of *T. p.* subsp. *pallidum* in serum samples from ambulatory patients from Rio de Janeiro, Brazil. A PRISMA-based systematic review was also performed to identify studies using molecular biology diagnosis from serum. Twenty serums screened by TPHA (Treponema pallidum Hemagglutination assay) and with different VDRL titers (Venereal Disease Research Laboratory test) were analyzed. Amplification of *tpp*15 gene was observed in 14/17 (82.35%) samples; *T. pallidum* sequence was confirmed in 12/17 (70.59%). The findings demonstrate the potential of molecular approaches based on aDNA-adapted protocols as alternatives to conventional serological diagnosis, contributing to improved detection of infection and strengthening epidemiological surveillance of syphilis.

## 1. Introduction

Syphilis remains a sexually transmitted and congenital infection of global relevance. Despite advances in diagnostic and therapeutic methods, the disease continues to have high incidence and prevalence in several regions worldwide, reflecting the need for epidemiological surveillance and prevention strategies [1,2,3]. *Treponema pallidum* subspecies *pallidum* belongs to the Spirochaetaceae family, characterized by its helical shape and high motility, which favor its dissemination in host tissues [4]. This subspecies is the etiological agent of syphilis, a primary sexually transmitted and congenital infection that represents a major concern for global public health. Syphilis is distinguished by its remarkable ability to mimic a variety of dermatological, neurological, and systemic manifestations, a characteristic that has earned it the classic title of “the great imitator” (*magna simulans*) [5].

The genus *Treponema* also includes other species and subspecies with different clinical presentations: *T. pallidum* subspecies *endemicum*, which causes bejel; *T. pallidum* subspecies *pertenue*, the etiological agent of yaws; and *T. carateum*, responsible for pinta. Despite sharing similar morphology, these bacteria differ in their modes of transmission and geographic distribution [6]. Syphilis progresses through well-defined clinical stages, shaped by the complex interaction between *T. pallidum* and the host immune response [7]. The primary stage typically presents with painless ulcers at the inoculation site, appearing two to three weeks after exposure [8]. Most untreated individuals progress to secondary syphilis, characterized by systemic symptoms and heterogeneous cutaneous lesions, frequently affecting palms and soles, an indicative feature of infection [9,10]. After resolution of these lesions, the agent may persist in a latent state and, without treatment, evolve into more severe forms with cardiovascular, neurological, or osseous complications [10].

Diagnostic strategies for syphilis include direct and indirect detection of *T. pallidum*. Direct methods include dark-field microscopy, silver staining, direct fluorescent antibody testing, and rabbit infectivity testing. Although classical, these techniques present important limitations, including variable sensitivity that depends on bacterial structure, the clinical stage of infection, and specimen quality [11]. Due to these challenges, serological tests, both treponemal and non-treponemal, remain the primary diagnostic tools. The VDRL (Venereal Disease Research Laboratory) and the RPR (Rapid Plasma Reagin) are widely used for screening and therapeutic follow-up. On the other hand, treponemal tests, such as TPHA (Treponema pallidum Hemagglutination Assay), FTA-ABS (Fluorescent Treponemal Antibody Absorption), and immune assays (EIA/ELISA), have higher specificity and are used for diagnostic confirmation [12,13].

Serological tests for syphilis are subject to false-positive and false-negative results, which may be related to the clinical stage of infection, the presence of co-infections, or the prozone effect [14,15,16]. Consequently, molecular biology techniques, particularly Polymerase Chain Reaction (PCR), have increasingly been used to directly detect *T. pallidum* DNA in various biological samples. However, the application of PCR in serum still presents significant challenges. The primary limitation is the low and variable bacterial load, due to transient spirochetemia [17,18]. Blood and serum contain natural inhibitors of PCR, such as proteins and lipids, which can interfere with the enzymatic amplification and reduce the sensitivity of the analysis [18]. On the other hand, samples from lesions or tissue exudates are considered more suitable for molecular detection of *T. pallidum*, as they have higher bacterial loads. A systematic review by Simpore and coauthors (2022) found a significant increase in the adoption of molecular biology methods between 2009 and 2019, with a predominance of conventional PCR and real-time PCR. Both performed satisfactorily, with consistently high specificity, although sensitivity varies with the clinical stage of the infection [19].

Ancient DNA (aDNA) studies have demonstrated the potential to detect *Treponema* spp. in archaeological materials, despite the challenges inherent to recovering fragmented DNA resulting from taphonomic processes [20,21,22,23]. To overcome these limitations, methodologies adapted to the analysis of highly degraded DNA or at low concentrations have been successfully applied [24]. Kolman and co-authors (1999) were the first to report the identification of *T. p.* ssp. *pallidum* in human remains from Easter Island, dated to approximately 240 ± 50 years BP, confirming infection in archaeological material [20]. Guedes and coauthors (2018) investigated the presence of *T. pallidum* in young individuals without signs of pathology, dated to the seventeenth and nineteenth centuries in Rio de Janeiro [21]. These results are part of a historical period marked by outbreaks of syphilis and yaws in the city, when it is estimated that about one-fifth of the population was infected [25]. These advances showed that, with optimized, sensitive protocols, it is possible to detect *T. pallidum* DNA even in highly degraded samples. In this context, the present study proposes applying a methodological protocol adapted from paleogenetic studies to detect *T. pallidum* in serological samples with low bacterial concentrations. Additionally, a PRISMA-guided systematic review was conducted to identify studies of molecular biology methods for syphilis diagnosis in serum samples, providing context for the interpretation of our findings. We hypothesize that the molecular detection of *T. p.* subsp. *pallidum* in serum samples can be achieved using protocols originally developed for archaeological DNA recovery.

## 2. Materials and Methods

### 2.1. Samples

Twenty serum samples from patients were analyzed, including sixteen with positive serology for syphilis, and as negative controls, two weakly reactive, and two non-reactive samples, as determined by VDRL and TPHA testing. Serological titers ranged from 1:2 to 1:8, and none of the individuals had a prior history of treatment. The samples were provided by the Laboratório de Diagnóstico, Ensino e Pesquisa (LADEP) of the Sergio Arouca National School of Public Health (ENSP/FIOCRUZ). Their use had been previously approved by the ENSP Research Ethics Committee under opinion No. 12/2013. Authorization to receive sera for molecular biology analyses was granted by the institution’s Scientific Committee, enabling the development of Guedes’ master’s thesis in 2014 [26]. The samples were subsequently stored in the biorepository of the Laboratório de Parasitologia Integrativa e Paleoparasitologia (LPIP/IOC/FIOCRUZ) for future investigations.

### 2.2. Molecular Biological Diagnostic Using aDNA Protocol

DNA extraction was carried out using the QIAamp DNA Mini Kit (Qiagen, Hilden, Germany), with protocol modifications designed to enhance aDNA recovery. For each sample, 200 μL of serum was processed, and a negative extraction control was included. In addition to the standard chemical digestion step, a physical lysis procedure was incorporated by exposing the samples to liquid nitrogen or dry ice, followed by vigorous vortex agitation at maximum speed to enhance cellular disruption. Centrifugation was performed at 14,000 rpm for 5 min, replacing the original protocol step of 8000 rpm for 2 min. Elution was conducted in a final volume of 40 μL to increase DNA concentration. DNA quantification was subsequently performed using the Quantus™ fluorometer (Promega, Madison, WI, USA).

A reconstructive polymerization pretreatment was applied to all extracted samples to facilitate the reconstruction and amplification of degraded DNA fragments, following the procedure described by Iñiguez in 2021 [24], a widely used technique in paleogenetic analyses. For the detection of *T. pallidum*, PCR amplification targeting the *tpp*15 gene was performed using the primers TL243F (5′-GAGCAGGATGTCTCTATGAGTTATAAAAGA-3′) and TH123R (5′-GAAGCCACTACCGATGTGCG-3′) [20], PCRs were set up in a final volume of 25 μL, containing 1× buffer, 1.75 mM MgCl_2_, 0.2 mM dNTPs, 10 μM of each primer, 3 U Platinum *Taq* DNA Polymerase (Promega), and 5 μL of extracted DNA (1–5 ng). The amplification protocol consisted of an initial denaturation at 95 °C for 5 min, followed by 50 cycles of denaturation at 95 °C for 40 s, annealing at 60 °C for 40 s, and extension at 72 °C for 40 s, with a final extension at 72 °C for 7 min. Reactions were carried out in a SimpliAmp™ thermocycler (Thermo Fisher Scientific, Waltham, MA, USA), and PCR negative controls were included to monitor for possible contamination.

The PCR products (pPCR) were analyzed by electrophoresis on a 2% agarose gel, stained with GelRed™ (Biotium, Fremont, CA, USA), and visualized under ultraviolet light using the Bio-Rad Transilluminator 2000 system (Hercules, CA, USA). Samples that did not yield visible amplicons were subjected to a second round of amplification under the same conditions used for the *tpp*15 marker. Positive amplicons were purified using the ExoSAP-IT™ reagent (Thermo Fisher Scientific, Waltham, MA, USA). DNA sequencing was performed with the BigDye™ Terminator v3.1 Cycle Sequencing Kit on the RPT01A DNA Sequencing Platform at FIOCRUZ, using the Applied Biosystems ABI 3730 sequencer (Foster City, CA, USA). The resulting sequences were processed and edited using BioEdit v5.0.9 and SeqMan v7.00 (DNASTAR Lasergene, Madison, WI, USA) and subsequently compared with reference sequences in the GenBank database via the NCBI/BLAST tool available at https://blast.ncbi.nlm.nih.gov/Blast.cgi, accessed on 12 July 2024.

In order to investigate the possible presence of inhibitors in the amplification reaction, the remaining negative samples were subjected to PCR using the 12S rDNA marker, widely used for the identification of vertebrates, as described by Kitano and coauthors (2008) [27]. The PCR with a final volume of 25 μL was performed with 1X buffer, 2 mM MgCl_2_, 0.2 mM dNTP, 10 μM each primer, 2U Platinum *Taq* DNA Polymerase (Promega, USA), and 5 µL or 1–10 ng/μL of DNA. The cycling included an initial cycle of 95 °C for 3 min, followed by 35 cycles of 30 s at 95 °C, 30 s at 57 °C, and 30 s at 72 °C with an extension of 10 min at 72 °C. The pPCR were analyzed as described above.

### 2.3. Comparative Evaluation of Ancient DNA and Conventional Molecular Protocols

To compare the ancient DNA protocol with a conventional molecular approach, PCR assays were performed with and without a reconstructive polymerization pre-treatment. The reconstructive polymerization pre-treatment is commonly employed in aDNA analyses prior to amplification. In parallel, PCR assays were conducted without this pre-treatment, following a conventional molecular workflow described above (Section 2.2).

### 2.4. Systematic Review

A systematic review framework was employed to identify studies that addressed the molecular biology diagnosis of syphilis in serum samples (Supplementary Figure S1).

## 3. Results

### 3.1. Molecular Biology Diagnosis of Syphilis in Serological Samples

The results revealed that 14 of 17 of the TPHA-positive or weakly reactive serum samples (82.35%) exhibited amplification of the *T. pallidum* marker. The presence of bacterial DNA was shown despite the low concentration of DNA obtained after the extraction step (Table 1). The TPHA-negative serum samples used as negative controls showed no *tpp*15 amplifications. Similarly, no amplification was detected in weakly reactive samples.

A total of 12/14 (85.71%) PCR-positive samples generated sequences that met quality criteria for comparison with the GenBank/NCBI database. The presence of *T. p.* subsp. *pallidum* was confirmed in 10/12 (83.33%) of the sequenced samples, based on the characteristic polymorphism (T191943C) of the subspecies (Supplementary Figure S2). Among the samples confirmed as *T. p.* subsp. *pallidum*, 10 presented serological titers of 1:2, 1:4, or 1:8 in the VDRL tests, showing concordance between serological and molecular data. However, in two samples (SR02 and SR13) with titers of 1:2 and 1:4, although amplification was successful and the sequences were of good quality, the region obtained did not include the segment corresponding to the differential polymorphism, limiting identification to the *T. pallidum* species level (Supplementary Figure S2). Two other samples (SR08 and SR12) with titers of 1:4 and 1:8 presented amplified products with the expected size, but not quality sequences. The sequences obtained were compromised by base noise, low signal intensity, and overlapping peaks in the electropherograms, making the comparative analysis with GenBank unfeasible. All negative samples showed 12S rDNA amplification with the molecular marker applied as an endogenous DNA control, indicating the integrity of the extracted DNA and the absence of inhibitory compounds that compromised the PCR activity.

### 3.2. Comparative Methodological Analysis

The comparative methodological analysis demonstrated differences in amplification efficiency between the conventional molecular protocol and the aDNA approach (Supplementary Figure S3). PCR assays performed without reconstructive polymerization pre-treatment yielded amplification in 7/18 samples (38.9%), indicating a lower detection rate when compared to assays preceded by reconstructive polymerization, which yielded amplifications in 14/18 samples (77.8%). In addition, PCR products obtained without reconstructive polymerization consistently exhibited weak low-intensity bands on agarose gels, which impairs the following sequencing procedures and consequently the confirmation of syphilis diagnosis. Electrophoretic profiles are provided in Supplementary Figure S3.

### 3.3. Systematic Review

Results of the systematic review showed a limited number of eligible studies addressing the molecular diagnosis of syphilis in serum samples, including four scientific articles and two master’s dissertations (Supplementary Table S1). The studies predominantly employed conventional PCR and real-time PCR targeting single-copy genes (Supplementary Table S1).

## 4. Discussion

In the present study, the *T. pallidum* detection rate was 82.35% TPHA seropositive individuals. Among the PCR-positive samples, 85.71% were identified as *T. p.* subsp. *pallidum* sequence. The serum samples were collected in 2013 and originally intended for the VDRL and TPHA serological tests of LADEP/FIOCRUZ. It is important to highlight that, even though these samples were stored for about 12 years, the application of aDNA recovery protocols enhanced the sensitivity of the PCR applied. The analysis not only enabled the detection of treponemal DNA in the serum samples but also provided insights into the limitations and potential of aDNA-based protocols within a contemporary clinical framework, particularly in samples with low bacterial loads.

The application of molecular methods in serological samples for the diagnosis of syphilis has been investigated since the end of the twentieth century [17,28]. Although the serum does not present the best performance for the direct detection of *T. pallidum*, due to the low concentration of circulating treponemas that hinder its lysis and DNA recovery [4,17], this material remains of great interest as it is widely used in clinical practice and often is the first sample collected during diagnostic investigation [29,30,31]. This availability underscores the importance of evaluating its potential in molecular approaches, although PCR analyses on serum samples are still infrequent. Nevertheless, conventional PCR-based molecular approaches are associated with higher costs and longer processing times, which may limit their routine application [31]. Molecular approaches enable more detailed epidemiological analyses, allowing the identification of circulating *T. pallidum* lineages (Nichols or SS14 clade) and the detection of genetic markers associated with antimicrobial resistance. Molecular surveillance is further justified by the increasing documentation of antimicrobial macrolide resistance in *T. pallidum*, associated with the 23S rRNA gene [32,33,34]. Evidence of reduced susceptibility or resistance to penicillin has also been documented [35,36,37]. The disease management relies primarily on penicillin G, while macrolides are commonly used in cases of penicillin allergy and have demonstrated efficacy as alternative therapeutic options [35,36,37,38].

Previous studies have reported detection rates in cases of primary and secondary syphilis in serological samples, ranging from 28.57% to 75% [28,39]. Martin and coauthors (2009) [31] reported that, in cases of primary syphilis, the molecular diagnosis performed on ulcer swabs or scrapings showed sensitivity close to 75%. On the other hand, in blood and serum samples from patients with secondary syphilis, positivity was approximately 50%. According to the authors, PCR performance is superior in primary lesions, where the bacterial load is higher and precedes complete seroconversion [31]. Blood and/or serum may be adequate when the bacteria is systemically disseminated. Durán Rodríguez and coauthors (2019) developed a qPCR targeting the *T. pallidum tpp*47 gene for the detection in serum from patients with suspected gestational and congenital syphilis, achieving 75% positivity and good agreement with serological tests [40]. Oliveira (2016) [41] conducted a molecular biology investigation involving patients with and without a history of HIV infection who were evaluated at the Souza Araújo Outpatient Clinic—FIOCRUZ. A number of 16/26 serum samples yielded *T. p.* subsp. *pallidum* sequences, and the A2058G mutation was detected in two of them, indicating the circulation of macrolide-resistant variants [41]. The detection of *T. p.* subsp. *pallidum* across multiple clinical specimen types, including saliva, serum, and CSF, was assessed in patients with primary, secondary, latent, and suspected neurosyphilis in China, totaling 1023 analyzed samples [42]. In serum, *T. p.* subsp. *pallidum* DNA was identified in 17 cases, with positivity concentrated in the secondary (2/59) and latent (10/412) stages, and no amplification was obtained from individuals with primary syphilis. Among patients with suspected neurosyphilis, 9.1% (5/55) of serum samples were positive. Overall, serum detection remained low (3.1%, 17/543), likely reflecting the limited circulating bacterial burden, which restricts the release of detectable nucleic acids into the bloodstream. The detection of treponemal DNA in individuals with latent syphilis suggests that this clinical stage may function as a persistent reservoir capable of sustaining transmission [42]. In the present study, the detection rates were higher than those reported in the literature identified through the PRISMA-based methodology.

The World Health Organization has established global strategic guidelines for the control and elimination of major public health challenges, including HIV, viral hepatitis, and other sexually transmitted infections (STIs), with particular emphasis on syphilis [43]. In the national context, Brazil reflects this epidemiological pattern: between 2010 and 2025, a total of 1,902,301 cases of acquired syphilis were reported [44]. In parallel, Brazilian public health strategies agenda for the elimination of congenital syphilis, establishing 2030 as a target year for its elimination through strengthened surveillance, expanded access to diagnosis and treatment [45,46,47].

The global response to syphilis involves stopping mother-to-child transmission, strengthening surveillance systems, timely diagnosis, and scaling up more accurate diagnostic tools capable of differentiating between active infection and specific manifestations, such as neurosyphilis and congenital syphilis [48]. In this context, molecular diagnostic testing is particularly relevant, as it allows confirmation of infection and monitoring of the therapeutic response. Despite the limited number of samples analyzed in the present study, we demonstrated the applicability of the molecular approach for serum diagnosis. Future investigations will include a larger number of samples to confirm the robustness of the approach presented here.

The present study has some limitations that should be considered when interpreting the results. First, clinical information regarding the disease status of patients, including the clinical stage of syphilis, was not available. The serum samples analyzed were archived samples originally collected at a single center (LADEP, ENSP/FIOCRUZ) and provided to our laboratory exclusively for use as control material in a paleogenetic study conducted in 2014 [25], under conditions that restricted access to associated clinical or epidemiological data. Consequently, limiting the evaluation of molecular detection rates in relation to disease stage. Second, the small number of samples analyzed may restrict the strength of the conclusions of the findings and preclude more detailed analyses across clinical or epidemiological subgroups. Third, the specificity of the assay was not systematically evaluated against a comprehensive panel of potential biological confounders or interfering agents. Although no false-positive results were observed in the samples analyzed. Nevertheless, samples that failed to yield *T. p.* subsp. *pallidum* amplifications were further evaluated for potential PCR inhibition. Finally, the use of long-term archived serum samples may have introduced uncontrolled pre-analytical variables affecting DNA integrity and detection efficiency.

Improving the supply of diagnostic methods, access to treatment, and the incorporation of prevention strategies in health services focused on acquired and congenital syphilis are essential. Strengthening molecular diagnosis by standardizing procedures, rigorously implementing quality control, and continuously training teams is important to ensure reliable results and support clinical decisions. In this scenario, the increasing application and dissemination of molecular biology techniques for STI diagnosis represent a major strength, as they promote technical consolidation, facilitate assay execution, and progressively reduce per-sample costs, thereby enhancing their feasibility for diagnostic and surveillance purposes.

In summary, the present study demonstrated an aDNA protocol as a reliable and accurate alternative for the diagnosis of active syphilis, showing the specific detection of *T. p.* subsp. *pallidum* polymorphism, so excluding *T. p.* subspecies *endemicum* (bejel), *T. p.* subspecies *pertenue* (yaws), and *T. carateum* (pinta); absence of false-positive results, which frequently occur in serological assays; and a level of detection in patients with serological titers 1:2–1:8, low values indicative, mainly, of early infections, but also recent latent syphilis (asymptomatic phase) suggesting possible active infection, which could allow a precocious treatment and follow-up until cure.

## Figures and Tables

**Table 1:** Results of molecular diagnosis of syphilis and data of serum samples from ambulatory patients, Rio de Janeiro, Brazil.

LPIP ID	VDRL Titers	TPHA	Extracted DNA Concentration (ng/µL)	Molecular Diagnostic Results
SR01	01:08	+	0.0349	*T. p.* subsp. *pallidum*
SR02	01:02	+	0.0538	*T. pallidum*
SR03	01:02	+	0.303	*T. p.* subsp. *pallidum*
SR04	01:02	+	0.0014	*T. p.* subsp. *pallidum*
SR05	01:08	+	0.0342	*T. p.* subsp. *pallidum*
SR06	01:04	+	0.0477	No Amplification
SR07	01:08	+	0.0362	*T. p.* subsp. *pallidum*
SR08	01:04	+	0.0714	Low-Quality Sequence
SR09	WR	WR	0.245	No Amplification
SR10	01:08	+	0.0858	*T. p.* subsp. *pallidum*
SR11	WR	WR	0.0537	No Amplification
SR12	01:08	+	0.0551	Low-Quality Sequence
SR13	01:04	+	0.250	*T. pallidum*
SR14	01:04	+	0.727	*T. p.* subsp. *pallidum*
SR15	01:02	+	0.0511	*T. p.* subsp. *pallidum*
SR16	01:04	+	0.0425	*T. p.* subsp. *pallidum*
SR17	01:02	+	0.266	*T. p.* subsp. *pallidum*
SR18	WR	−	0.0144	No Amplification
SR19	NR	−	0.0070	No Amplification
SR20	NR	−	0.103	No Amplification

Serological results WR: Weakly Reactive; NR: Non-Reactive; +: Positive; −: Negative.

## Data Availability

The original contributions presented in this study are included in the article/Supplementary Materials. Further inquiries can be directed to the corresponding author.

## Supplementary Materials

The following supporting information can be downloaded at https://www.mdpi.com/article/10.3390/microorganisms14020453/s1. Figure S1. PRISMA 2020 flow diagram illustrates the identification, screening, eligibility assessment, and inclusion of studies in the systematic review. Figure S2. Sequence Alignment of *T. p.* subsp. *pallidum tpp*15 Amplicons Generated in the Present Study. Figure S3. Electrophoresis Gels of PCR amplification of the *T. p.* subsp. *pallidum tpp*15 target with and without the application of ancient DNA protocols. Table S1. Systematic Review Results of Studies Evaluating the Molecular Diagnosis of Syphilis in Serum. Reference [49] is cited in the Supplementary Figure S1.

## Abbreviations

The following abbreviations are used in this manuscript:

aDNA	ancient DNA
BP	Before Present
BLAST	Basic Local Alignment Search Tool
CSF	Cerebrospinal Fluid
cPCR	Convencional PCR
DNA	Deoxyribonucleic acid
EIA	Enzyme Immunoassay
ELISA	Enzyme-Linked Immunosorbent Assay
ENSP	Escola Nacional de Saúde Pública Sergio Arouca
FIOCRUZ	Fundação Oswaldo Cruz
FTA-ABS	Fluorescent Treponemal Antibody Absorption
HIV	Human Immunodeficiency Virus
IOC	Instituto Oswaldo Cruz
LADEP	Laboratório de Diagnóstico, Ensino e Pesquisa
LPIP	Laboratório de Parasitologia Integrativa e Paleoparasitologia
mPCR	Multiplex PCR
NCBI	National Center for Biotechnology Information
nPCR	Nested PCR
PAHO	Pan American Health Organization
PCR	Polymerase Chain Reaction
qPCR	Quantitative PCR
rDNA	Deoxyribonucleic acid ribossomal
STIs	Sexually Transmitted Infections
TPHA	*Treponema pallidum* Hemagglutination Assay
VDRL	Venereal Disease Research Laboratory
WHO	World Health Organization

## References

[1] Kojima N., Klausner J.D. (2018). An Update on the Global Epidemiology of Syphilis. Curr. Epidemiol. Rep..

[2] Tao Y.-T., Gao T.-Y., Li H.-Y., Ma Y.-T., Li H.-J., Xian-Yu C.-Y., Deng N.-J., Zhang C. (2023). Global, Regional, and National Trends of Syphilis from 1990 to 2019: The 2019 Global Burden of Disease Study. BMC Public Health.

[3] Rosset F., Celoria V., Delmonte S., Mastorino L., Sciamarrelli N., Boskovic S., Ribero S., Quaglino P. (2025). The Epidemiology of Syphilis Worldwide in the Last Decade. J. Clin. Med..

[4] LaFond R.E., Lukehart S.A. (2006). Biological Basis for Syphilis. Clin. Microbiol. Rev..

[5] Osler W. (1902). The Principles and Practice of Medicine: Designed for the Use of Practitioners and Students of Medicine.

[6] Pla-Diaz M., Giacani L., Tantalo L.C., Bose M., Reid T.B., Marra C.M., Šmajs D., Pospíšilová P., Janečková K., Kawahata T. (2025). A New Typing Scheme Demonstrates High Discriminatory Power for *Treponema pallidum* Subspecies. bioRxiv.

[7] Kent M.E., Romanelli F. (2008). Reexamining Syphilis: An Update on Epidemiology, Clinical Manifestations, and Management. Ann. Pharmacother..

[8] Chaudhry S., Akinlusi I., Shi T., Cervantes J. (2023). Secondary Syphilis: Pathophysiology, Clinical Manifestations, and Diagnostic Testing. Venereology.

[9] Stamm L.V. (2016). Syphilis: Re-Emergence of an Old Foe. Microb. Cell.

[10] Zhou S., Chanderraj R. (2023). What Is Syphilis?. JAMA.

[11] Zhou C., Zhang X., Zhang W., Duan J., Zhao F. (2019). PCR Detection for Syphilis Diagnosis: Status and Prospects. J. Clin. Lab. Anal..

[12] Young H. (2000). Guidelines for Serological Testing for Syphilis. Sex. Transm. Infect..

[13] Sáez-Alquézar A., Albieri D., Garrini R.H.C., Marques W.P., de Lemos E.A., Alves A. (2007). Desempenho de testes sorológicos para sífilis, treponêmicos (ELISA) e não treponêmicos (VDRL e RPR), na triagem sorológica para doadores de sangue—Confirmação dos resultados por meio de três testes treponêmicos (FTA ABS, WB E TPHA). Rev. Patol. Trop./J. Trop. Pathol..

[14] Soreng K., Levy R., Fakile Y. (2014). Serologic Testing for Syphilis: Benefits and Challenges of a Reverse Algorithm. Clin. Microbiol. Newsl..

[15] Nieuwenburg S.A., Zondag H.C.A., Bruisten S.M., Jongen V.W., Schim van der Loeff M.F., van Dam A.P., de Vries H.J.C. (2022). Detection of *Treponema pallidum* DNA During Early Syphilis Stages in Peripheral Blood, Oropharynx, Ano-Rectum and Urine as a Proxy for Transmissibility. Clin. Infect. Dis..

[16] Potekaev N., Zhukova O., Khamaganova I., Potekaev N., Zhukova O., Khamaganova I. (2022). False-Positive Serologic Reactions for Syphilis. Bacterial Sexually Transmitted Infections—New Findings, Diagnosis, Treatment, and Prevention.

[17] Grimprel E., Sanchez P.J., Wendel G.D., Burstain J.M., McCracken G.H., Radolf J.D., Norgard M.V. (1991). Use of Polymerase Chain Reaction and Rabbit Infectivity Testing to Detect *Treponema pallidum* in Amniotic Fluid, Fetal and Neonatal Sera, and Cerebrospinal Fluid. J. Clin. Microbiol..

[18] Grange P.A., Gressier L., Dion P.L., Farhi D., Benhaddou N., Gerhardt P., Morini J.P., Deleuze J., Pantoja C., Bianchi A. (2012). Evaluation of a PCR Test for Detection of *Treponema pallidum* in Swabs and Blood. J. Clin. Microbiol..

[19] Simpore A., Bazie B.V., Zoure A.A., Ouattara A.K., Compaore R.T., Kiba-Koumare A., Yooda P.A., Djigma F.W., Sombié H.K., Bisseye C. (2022). Performance of Molecular Tests in the Diagnosis of Syphilis From 2009 to 2019: A Systematic Review and Meta-Analysis. Sex. Transm. Dis..

[20] Kolman C.J., Centurion-Lara A., Lukehart S.A., Owsley D.W., Tuross N. (1999). Identification of *Treponema pallidum* subspecies *pallidum* in a 200-Year-Old Skeletal Specimen. J. Infect. Dis..

[21] Guedes L., Dias O., Neto J., Ribeiro da Silva L.d.P., Mendonça de Souza S.M.F., Iñiguez A.M. (2018). First Paleogenetic Evidence of Probable Syphilis and Treponematoses Cases in the Brazilian Colonial Period. BioMed Res. Int..

[22] Giffin K., Lankapalli A.K., Sabin S., Spyrou M.A., Posth C., Kozakaitė J., Friedrich R., Miliauskienė Ž., Jankauskas R., Herbig A. (2020). A Treponemal Genome from an Historic Plague Victim Supports a Recent Emergence of Yaws and Its Presence in 15th Century Europe. Sci. Rep..

[23] Montiel R., Solórzano E., Díaz N., Álvarez-Sandoval B.A., González-Ruiz M., Cañadas M.P., Simões N., Isidro A., Malgosa A. (2012). Neonate Human Remains: A Window of Opportunity to the Molecular Study of Ancient Syphilis. PLoS ONE.

[24] Iñiguez A.M., Shin D.H., Bianucci R. (2021). Ancient DNA and Paleoparasitology in Brazil. The Handbook of Mummy Studies New Frontiers in Scientific and Cultural Perspectives.

[25] Ribeiro B.V.D., Galdencio R., Pinto E.E.P., Drumond S., de Oliveira L.M.C. (2021). Um século de sífilis no Brasil: Deslocamentos e aproximações das campanhas de saúde de 1920 e 2018/2019. Rev. Bras. Hist. Mídia.

[26] Guedes L. (2014). Análise Paleogenética de Treponemas Em Remanescentes Humanos Do Período Histórico Brasileiro (Séculos XVII Ao XIX). Master’s Thesis.

[27] Kitano T., Umetsu K., Tian W., Osawa M. (2007). Two Universal Primer Sets for Species Identification among Vertebrates. Int. J. Leg. Med..

[28] Pietravalle M., Pimpinelli F., Maini A., Capoluongo E., Felici C., D’Auria L., Di Carlo A., Ameglio F. (1999). Diagnostic Relevance of Polymerase Chain Reaction Technology for *T. pallidum* in Subjects with Syphilis in Different Phases of Infection. New Microbiol..

[29] Young H. (1998). SYPHILIS: Serology. Dermatol. Clin..

[30] Morshed M.G., Adhikari R., Thapa S. (2014). Current Trend on Syphilis Diagnosis: Issues and Challenges. Proceedings of the Infectious Diseases and Nanomedicine II.

[31] Martin I.E., Tsang R.S.W., Sutherland K., Tilley P., Read R., Anderson B., Roy C., Singh A.E. (2009). Molecular Characterization of Syphilis in Patients in Canada: Azithromycin Resistance and Detection of *Treponema pallidum* DNA in Whole-Blood Samples versus Ulcerative Swabs. J. Clin. Microbiol..

[32] Eguiluz M., Vargas S., Giacani L., Reyes-Diaz M., Konda K., Caceres C., Klausner J. (2021). P229 Prevalence of Macrolide and Tetracycline Resistant *Treponema pallidum* Strains in Syphilis Cases, Lima and Pucallpa, Peru. Sex. Transm. Infect..

[33] Wang X., Abliz P., Deng S. (2023). Molecular Characteristics of Macrolide Resistance in *Treponema pallidum* from Patients with Latent Syphilis in Xinjiang, China. Infect. Drug Resist..

[34] Li Z., Hou J., Zheng R., Li Z., Wen J., Liu D., Liu R., Chu T., Liu B., Yu G. (2020). Two Mutations Associated with Macrolide Resistance in *Treponema pallidum* in Shandong, China. J. Clin. Microbiol..

[35] Jaiswal A.K., Gomes L.G.R., de Oliveira A.F.M., Soares S.d.C., Azevedo V., Jaiswal A.K., Gomes L.G.R., de Oliveira A.F.M., Soares S.d.C., Azevedo V. (2025). The Critical Role of Penicillin in Syphilis Treatment and Emerging Resistance Challenges. Diseases.

[36] Mi H.-F., Shen X., Chen X.-Q., Zhang X.-L., Ke W.-J., Xiao Y. (2023). Association between Treatment Failure in Patients with Early Syphilis and Penicillin Resistance-Related Gene Mutations of *Treponema pallidum*: Protocol for a Multicentre Nested Case–Control Study. Front. Med..

[37] Pospíšilová P., Bosák J., Hrala M., Krbková L., Vrbová E., Šmajs D. (2025). Resistance to Ceftriaxone and Penicillin G among Contemporary Syphilis Strains Confirmed by Natural in Vitro Mutagenesis. Commun. Med..

[38] Giacani L. (2025). Antibiotic Resistance in *Treponema pallidum* subsp. *pallidum*, the Syphilis Agent. Braz. J. Sex. Transm. Dis..

[39] Kouznetsov A.V., Weisenseel P., Trommler P., Multhaup S., Prinz J.C. (2005). Detection of the 47-Kilodalton Membrane Immunogen Gene of *Treponema pallidum* in Various Tissue Sources of Patients with Syphilis. Diagn. Microbiol. Infect. Dis..

[40] Durán-Rodriguez A.T., Navarrete-Ospina J., Muñoz-Molina L.C., Chavarro-Portillo B., Arenas-Moreno L., Pinilla-Bermúdez G. (2020). Molecular Detection of Gestational and Congenital Syphilis. Infectio.

[41] Oliveira P.C. (2016). Sífilis: Diagnóstico e Identificação Molecular de Amostras Clínicas de Pacientes Com Apresentação Atípica. Master’s Thesis.

[42] Meng Y., Yang L., Fu Y., Li S., Hamal K., Liu D. (2025). Detection of *Treponema pallidum tpp*47 DNA in Clinical Samples of Syphilis Patients. Eur. J. Med. Res..

[43] World Health Organization New Report Flags Major Increase in Sexually Transmitted Infections, amidst Challenges in HIV and Hepatitis. https://www.who.int/news/item/21-05-2024-new-report-flags-major-increase-in-sexually-transmitted-infections---amidst-challenges-in-hiv-and-hepatitis.

[44] Boletim Epidemiológico de Sífilis (2025). Departamento de HIV, Aids, Tuberculose, Hepatites Virais e Infecções Sexualmente Transmissíveis (Dathi), da Secretaria de Vigilância em Saúde e Ambiente (SVSA) do Ministério da Saúde (MS).

[45] Ministério da Saúde Saúde Reforça Ações de Combate à Sífilis e Mira na Eliminação da Doença Até 2030. https://www.gov.br/saude/pt-br/assuntos/noticias/2024/outubro/saude-reforca-acoes-de-combate-a-sifilis-e-mira-na-eliminacao-da-doenca-ate-2030.

[46] Passos M.R.L. (2023). Eliminação da sífilis congênita: Dever de todos. J. Bras. Ginecol..

[47] Pan American Health Organization Guidance for the Elimination of Syphilis and Congenital Syphilis in the Americas. https://iris.paho.org/handle/10665.2/61824.

[48] Miranda A.E., Freitas F.L.S., de Passos M.R.L., Lopez M.A.A., Pereira G.F.M. (2021). Políticas públicas em infecções sexualmente transmissíveis no Brasil. Epidemiol. Serviços Saúde.

[49] Page M.J., McKenzie J.E., Bossuyt P.M., Boutron I., Hoffmann T.C., Mulrow C.D., Shamseer L., Tetzlaff J.M., Akl E.A., Brennan S.E. (2021). The PRISMA 2020 statement: An updated guideline for reporting systematic reviews. BMJ.

