# Supplementary material for: Molecular Diagnosis of Syphilis in Brazilian Ambulatory Patients: Detection of Treponema pallidum subsp. pallidum in Serum Using Ancient DNA Protocols
Source: Microorganisms. 2026 Feb 12;14(2):453. doi: 10.3390/microorganisms14020453 (PMC12942721; doi:10.3390/microorganisms14020453)
Supplement: Supplementary file 1 [file microorganisms-14-00453-s001.zip › Table S1 - Systematic Review Results.pdf]

Table S1. Systematic Review Results of Studies Evaluating the Molecular Diagnosis of Syphilis in Serum.

Authors	Type of Publication	Country	Nº Samples	Molecular Biology Technique	Target Gene	Positive Diagnostic Outcomes
Durán-Rodriguez et al., 2019	Scientific article	Colombia	100	qPCR	*tpp*47	44 (44%)
Guedes, 2014	Master's dissertation	Brazil	18	cPCR	*tpp*15	12 (66,7)
Grimprel et al., 1991	Scientific article	United States	12	cPCR	*tpp*47	9 (75%)
Kouznetsov et al., 2004	Scientific article	Germany	7	nPCR	*tpp*47	2 (28,57%)
Oliveira., 2016	Master's dissertation	Brazil	24	mPCR	*tpp*15	16 (66,7%)
Meng et al., 2025	Scientific article	China	543	nPCR	*tpp*47	17 (3.1%)

cPCR: Convencional PCR; qPCR: Quantitative PCR; mPCR: Multiplex PCR; nPCR: Nested PCR; Nº: Number.

From the search strategies applied across databases, a total of 261 publications related to the topic were identified using the set of specific terms defined in the methodology. During the subsequent screening and selection stages, duplicates and studies that did not meet the eligibility criteria were progressively removed in accordance with the parameters established by the PRISMA 2020 flow diagram model. Following the review of titles, abstracts, and full texts, four scientific articles and two dissertations fulfilled the inclusion criteria and were selected for this study. The included publications directly addressed the molecular biology diagnosis of syphilis in serum samples, employing molecular biology techniques such as conventional PCR and real-time PCR, with varying approaches to sensitivity, specificity, and comparison with traditional serological methods. In line with the objectives of the PRISMA methodology, a summary table was compiled presenting the principal information extracted from each study, including authorship, year of publication, country, sample type, molecular technique, target gene, and diagnostic outcomes (Table S1). This systematization enabled a comparative analysis of molecular biology diagnostic strategies for syphilis and helped identify both methodological gaps and potential strengths of these approaches for serological samples.
