# Supplementary material for: Molecular Diagnosis of Syphilis in Brazilian Ambulatory Patients: Detection of Treponema pallidum subsp. pallidum in Serum Using Ancient DNA Protocols
Source: Microorganisms. 2026 Feb 12;14(2):453. doi: 10.3390/microorganisms14020453 (PMC12942721; doi:10.3390/microorganisms14020453)
Supplement: Supplementary file 1 [file microorganisms-14-00453-s001.zip › Figure S2 - Sequence Alignment tpp15.pdf]

Figure S2. Sequence Alignment of *T. p.* subsp. *pallidum tpp15* Amplicons Generated in the Present Study.

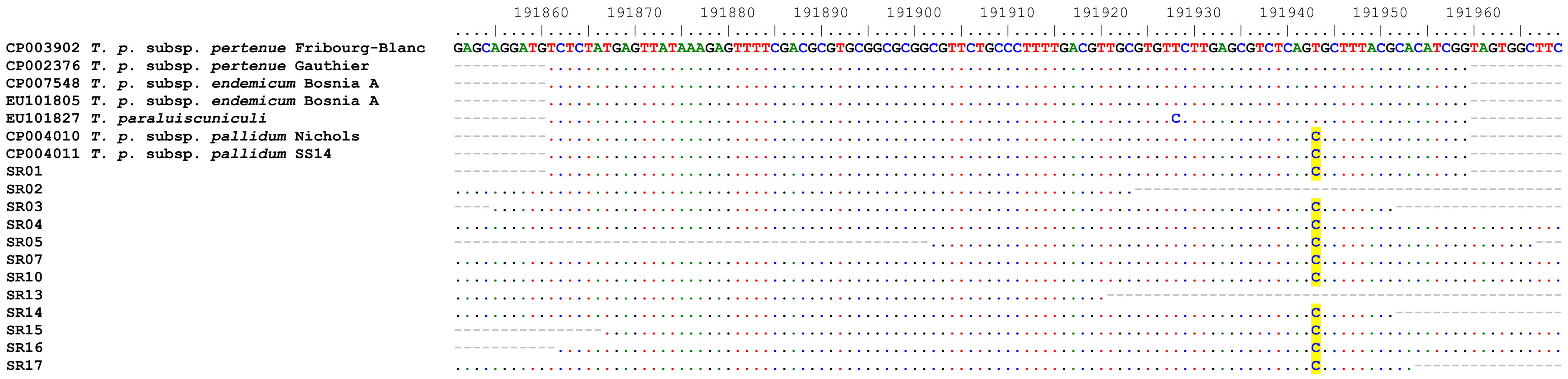

The reference genome is *T. p.* subsp. *pertenue* Fribourg-Blanc (CP003902) and representative sequences from other *Treponema* taxa are included. The polymorphism characteristic of *T. p.* subsp. *pallidum* (T191943C) is highlighted.
