# Supplementary material for: Molecular Diagnosis of Syphilis in Brazilian Ambulatory Patients: Detection of Treponema pallidum subsp. pallidum in Serum Using Ancient DNA Protocols
Source: Microorganisms. 2026 Feb 12;14(2):453. doi: 10.3390/microorganisms14020453 (PMC12942721; doi:10.3390/microorganisms14020453)
Supplement: Supplementary file 1 [file microorganisms-14-00453-s001.zip › Figure S3 - Electrophoresis Gels.pdf]

Figure S3. Electrophoresis Gels of PCR amplification of the *T. p.* subsp. *pallidum tpp*15 target with and without the application of ancient DNA protocols.

Electrophoresis gels obtained using the ancient DNA protocol are shown in Figures I, II, and III whereas gels generated using the conventional molecular protocol, without the application of ancient DNA procedures, are presented in Figures IV and V.

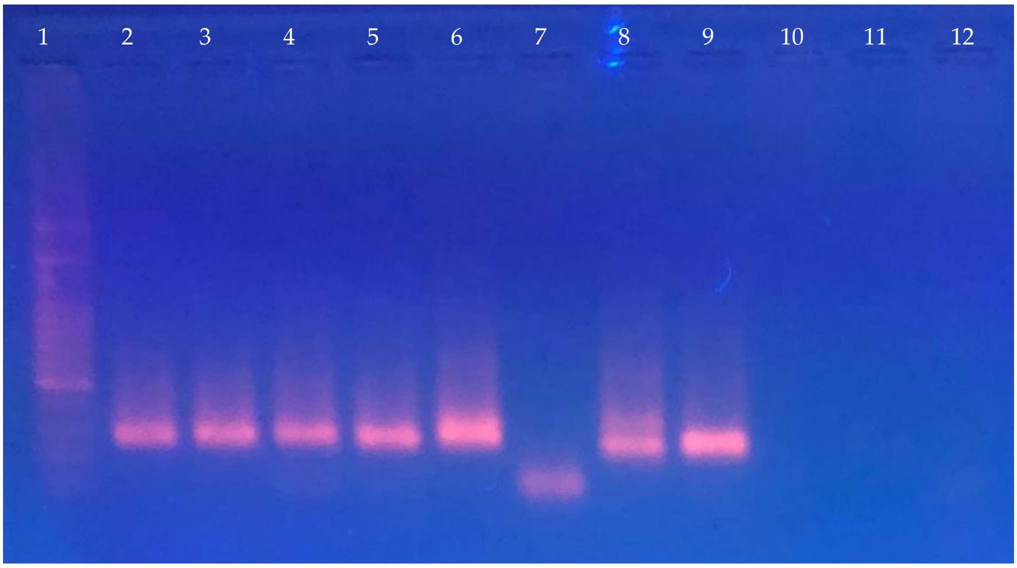

I. Results of 3% agarose gel electrophoresis of PCR amplification targeting the *tpp*15 gene from serum samples. Lane 1: 50 bp DNA ladder (Ludwig). Lanes 2–10: SR01, SR02, SR03, SR04, SR05, SR06, SR07, SR08, and SR09. Lane 11: empty. Lane 12: PCR negative controls. Expected amplicon size: 120 bp.

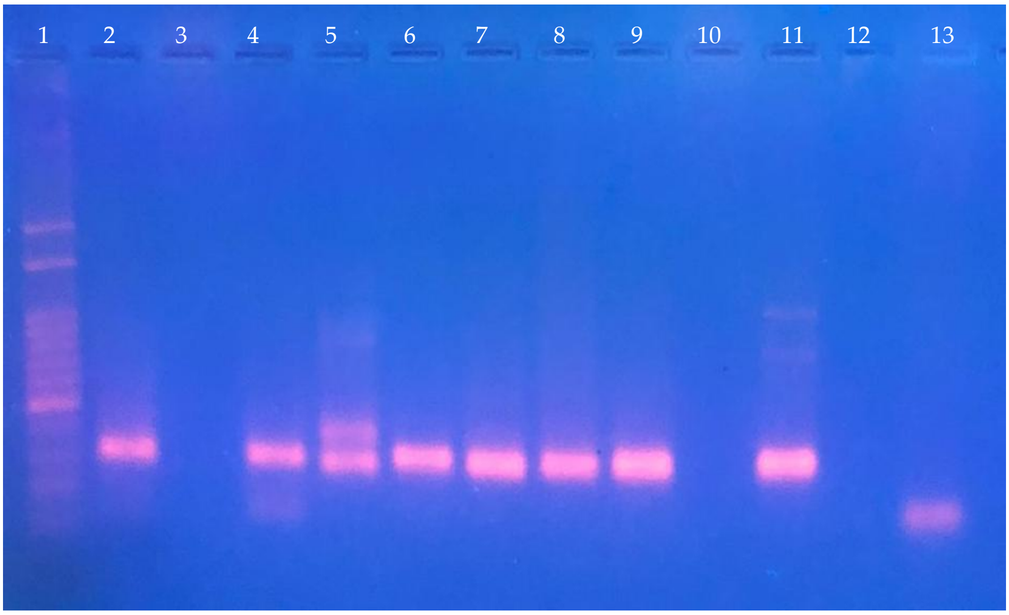

II. Results of 3% agarose gel electrophoresis of PCR amplification targeting the *tpp*15 gene from serum samples. Lane 1: 50 bp DNA ladder (Ludwig). Lanes 2–10: SR10, SR11, SR12, SR13, SR14, SR15, SR16, SR17, and SR18. Lane 11: positive control (CDC strain). Lane 12: empty. Lane 13: PCR negative controls. Expected amplicon size: 120 bp.

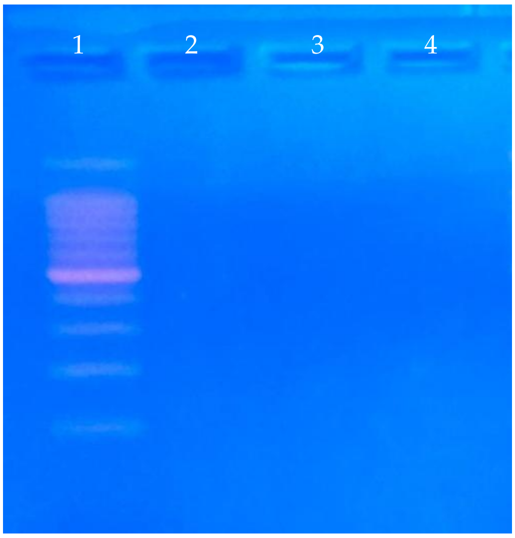

III. Results of 3% agarose gel electrophoresis of PCR amplification targeting the *tpp*15 gene from serum samples. Lane 1: 50 bp DNA ladder (Ludwig). Lanes 2–3: SR19 and SR20. Lane 4: PCR negative controls. Expected amplicon size: 120 bp.

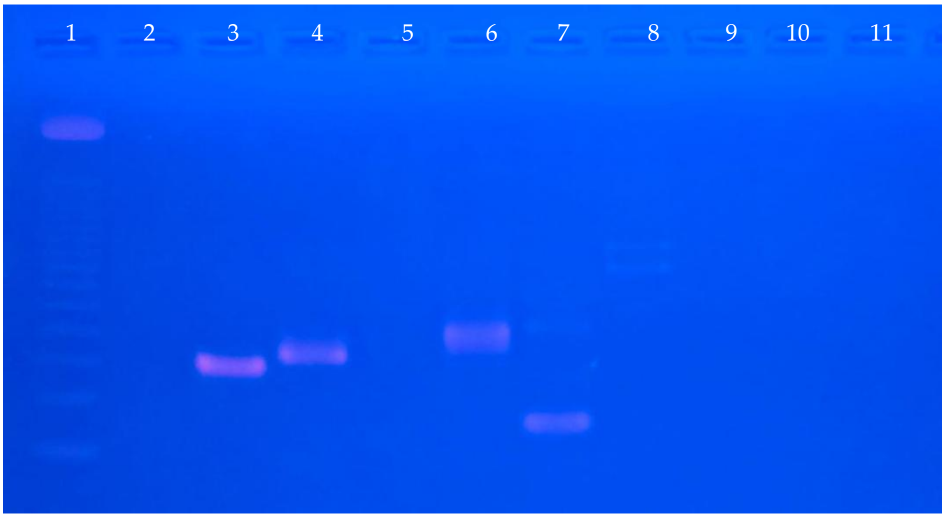

IV. Results of 3% agarose gel electrophoresis of PCR amplification targeting the *tpp*15 gene from serum samples. Lane 1: 50 bp DNA ladder (Ludwig). Lanes 2–10: SR01, SR02, SR03, SR04, SR05, SR06, SR07, SR08, and SR09. Lane 11: PCR negative controls. Expected amplicon size: 120 bp.

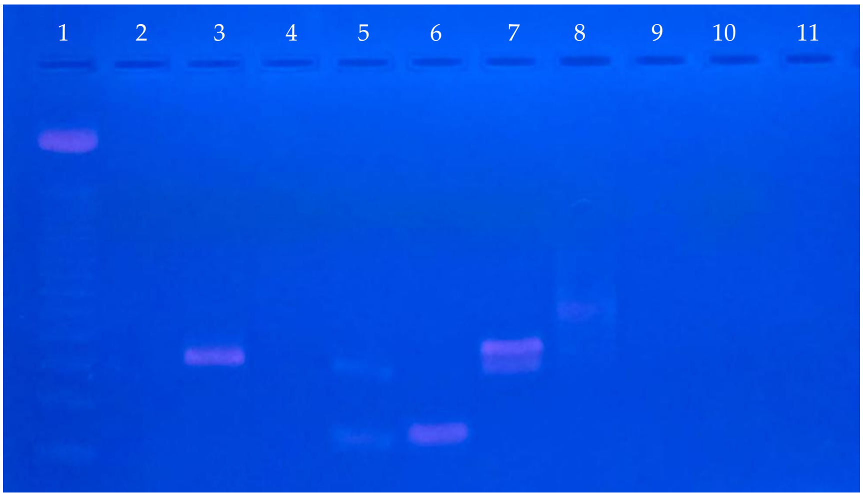

V. Results of 3% agarose gel electrophoresis of PCR amplification targeting the *tpp*15 gene from serum samples. Lane 1: 50 bp DNA ladder (Ludwig). Lanes 2–10: SR10, SR11, SR12, SR13, SR14, SR15, SR16, SR17, and SR18. Lane 11: PCR negative controls. Expected amplicon size: 120 bp.
